# Therapy-resistant nature of cancer stem cells in view of iron metabolism

**DOI:** 10.1186/s41232-022-00220-y

**Published:** 2022-11-03

**Authors:** Wenqian Wang, Kouichi Tabu, Alapati Aimaitijiang, Tetsuya Taga

**Affiliations:** 1 Department of Plastic Surgery, The Second Affiliated Hospital and Yuying Children’s Hospital of Wenzhou Medical University, Wenzhou, China; 2 Department of Stem Cell Regulation, Medical Research Institute, Tokyo Medical and Dental University (TMDU), 1-5-45, Yushima, Bunkyo-ku, Tokyo, 113-8510 Japan

**Keywords:** Cancer stem cell, Iron metabolism, Iron chelator, Nanotechnology, Ferroptosis, 5-ALA-PDT

## Abstract

Due to increased resistance to standard chemo/radiotherapies and relapse, highly tumorigenic cancer stem cells (CSCs) have been proposed as a promising target for the development of effective cancer treatments. In order to develop innovative cancer therapies that target CSCs, much attention has focused on the iron metabolism of CSCs, which has been considered to contribute to self-renewal of CSCs. Here, we review recent advances in iron metabolism and conventional iron metabolism-targeted cancer therapies, as well as therapy resistance of CSCs and potential treatment options to overcome them, which provide important insights into therapeutic strategies against intractable cancers. Potential treatment options targeting iron homeostasis, including small-molecule inhibitors, nanotechnology platforms, ferroptosis, and 5-ALA-PDT, might be a focus of future research for the development of innovative cancer therapies that tackle CSCs.

## Background

Due to increased resistance to standard chemo/radiotherapies and relapse, highly tumorigenic cancer stem cells (CSCs) have been proposed as a promising target for the development of effective cancer treatments [1]. Iron is critical for cancer progression and recurrence, and a variety of iron metabolism-related proteins is abnormally regulated in CSCs, implicating that an augmented accumulation of intracellular iron and iron-dependency are one of metabolic hallmarks of CSCs [2]. In order to develop innovative cancer therapies that target CSCs, much attention has focused on the iron metabolism of CSCs, which has been considered to be important for self-renewal of CSCs [3–5]. Thus, targeting iron metabolic pathways in CSCs represents a valuable strategy which impairs therapy-resistant nature of CSCs and thereby inhibiting the cancer recurrence. However, resistant mechanisms of CSCs on iron metabolism-targeted cancer therapy remain to be fully elucidated. Here, we review the mechanisms underlying therapy resistance of CSCs particularly by focusing on the iron metabolism and potential strategies to overcome them.

## Main text

### Iron metabolism in CSCs and non-CSCs

**Fig. 1:**
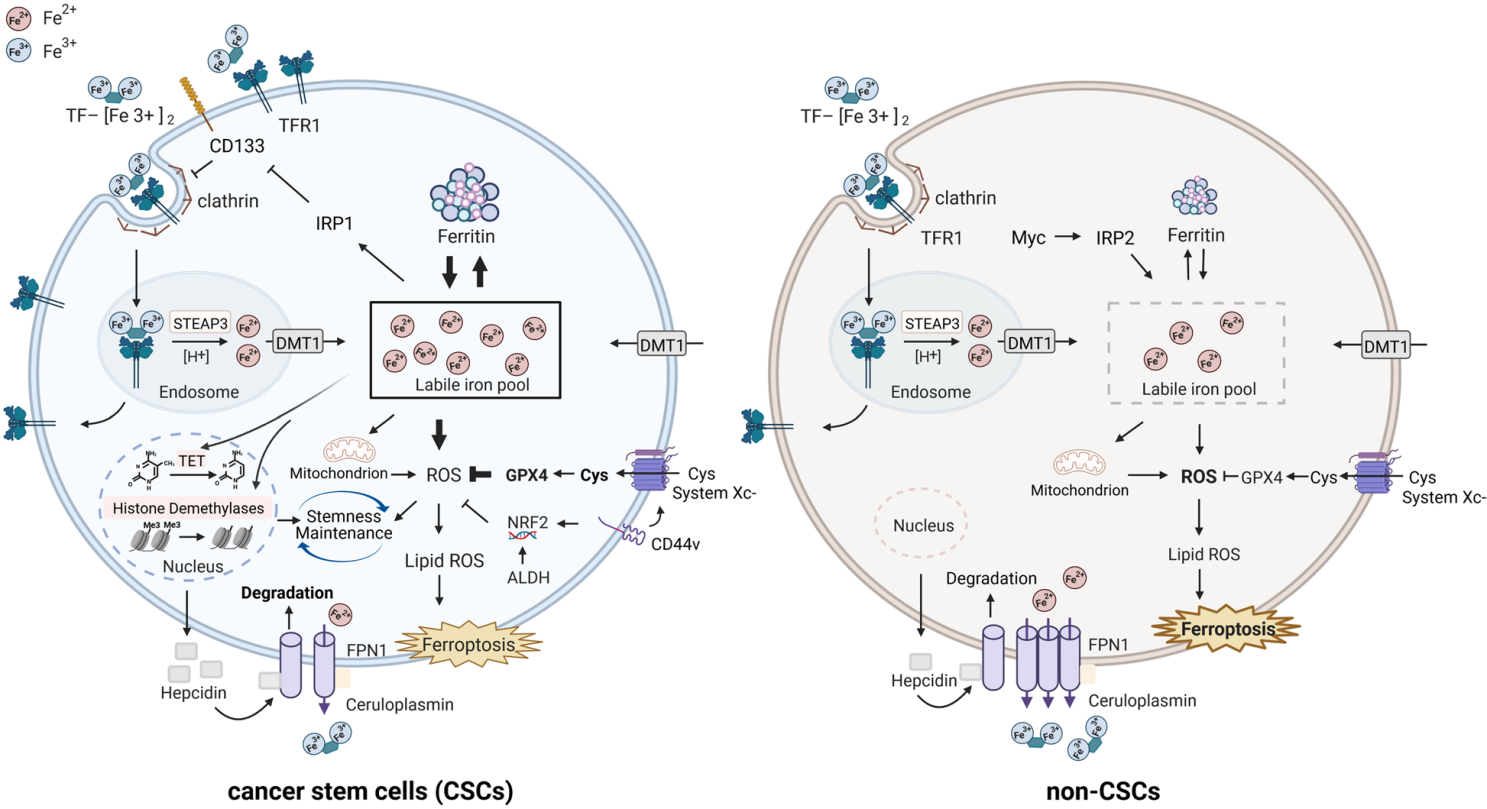
Iron metabolism in cancer stem cells (CSCs) and non-CSCs. CSCs and non-CSCs differently express proteins implicated in iron trafficking. CSCs possess an augmented accumulation of intracellular iron and iron-dependent maintenance and expansion via ROS and epigenetic reprogramming, resulting in therapy resistance. Moreover, compared to non-CSCs, CSCs possess nature resistance of rendering them sensitive to ferroptosis. ALDH, aldehyde dehydrogenase; CD44v, CD44 variant isoform; Cys, cystine; DMT1, divalent metal transporter 1; TF, transferrin; TFR1, transferrin receptor 1; FPN1, ferroportin 1; GPX4, glutathione peroxidase 4; IRP1/2, iron regulatory protein 1/2; NRF2, nuclear factor erythroid 2-related factor 2; ROS, reactive oxygen species; System Xc-, cystine/glutamic acid transporter; STEAP3, six epithelial transmembrane antigensof the prostate 3

Dysregulated iron homeostasis has been considered as a metabolic hallmark of CSCs, in which some alterations of iron trafficking have been identified compared to cancer cells (Fig. 1) [6]. These changes clearly indicated that an augmented accumulation of intracellular iron is associated with iron-dependent maintenance and expansion of CSCs, resulting in therapy resistance of bulk tumors [2]. Transferrin (TF) binding to transferrin receptor 1 (TFR1) is a major starting point of the way to increase iron uptake [7]. Recent studies revealed that TF protein is upregulated in glioblastoma and ovarian CSCs than non-CSCs [3, 8]. It has been observed that expression levels of *Tfr1* gene are increased in the CSCs of breast, prostate, and cholangiocarcinoma, suggesting that CSCs exhibit an enhanced iron uptake [4, 8, 9]. Ferroportin 1 (FPN1) is the sole cellular iron exporter, and hepcidin induces FPN1 internalization and degradation, thereby inhibiting iron efflux [6]. The expression levels of *Fpn1* gene are downregulated in ovarian and cholangiocarcinoma CSCs [3, 10]. There is increasing evidence indicating that patients of multiple tumor types have high levels of serum and tumor hepcidin, although the levels of hepcidin in CSCs remain to be fully investigated [11, 12]. Ceruloplasmin, a ferroxidase involved in iron transport that facilitates iron flux from cells, is found to be reduced in CSCs as an additional mechanism of iron retention [7]. Along with internalization of iron-TF by endocytosis, iron is reduced by six-transmembrane epithelial antigen of the prostate 3 (STEAP3) in the endosome and is subsequently exported by divalent metal transporter 1 (DMT1) into the cytoplasmic labile iron pool (LIP). Cellular iron homeostasis is post-transcriptionally regulated by iron regulatory proteins, IRP1 and IRP2, which are RNA-binding proteins that bind to iron-responsive elements (IREs) of the major proteins of iron metabolism [13]. LIP is enhanced in non-CSCs by upregulation of IRP2, which is regulated by proto-oncogene and proliferation gene c-Myc [14]. IRP1, which is the regulator of iron metabolism-related gene expression, binds to IREs of some stem cell genes including CD133 to inhibit their translation or degradation [15]. CSCs have enhanced iron uptake and reduced iron efflux, which collectively lead to high intracellular iron. A forced increase in intracellular iron promotes metastatic spread of ovarian CSCs through the expression of matrix metalloproteases and synthesis of IL-6 [16]. The majority of cytoplasmic iron is stored in ferritin, which increases in glioblastoma stem-like cells compared to non-CSCs [3]. Recent studies show that high heavy-chain ferritin (H-ferritin) expression seems to relate closely with CSC features in breast and cholangiocarcinomas [8, 9]. However, except for TFR1, levels of proteins involved in iron trafficking are still disputed between CSCs and non-CSCs in different cancers (Fig. 1). The CSC addiction to iron might result in part from the role of iron in stemness maintenance, as iron activates self-renewal and influences the differentiation status of CSCs via mitochondrial reactive oxygen species (ROS) and epigenetic reprogramming such as DNA and histone demethylation [6, 17]. The important role of iron in stem cell dynamics is its essential function as a co-factor of epigenetic enzymes including TET enzymes and JmjC domain-containing proteins. CSCs alter the canonical Notch, Wnt, and hedgehog signaling pathways for CSC maintenance and self-renewal ability by iron-mediated epigenetic mechanisms [18, 19]. Taken together, some of the major proteins in iron metabolism might have therapeutic potential for CSC treatment, such as iron transporter TF and its receptor, iron storage protein ferritin, iron exporter FPN1, iron regulator hepcidin, and epigenetic enzymes.

### Current cancer treatments targeting iron metabolism

Recent studies depicting the molecular characterization of alterations of iron homeostasis aimed at maintaining the high iron demand of CSCs [20] offered support to the use of iron chelators as anti-tumor drugs [21]. Iron chelators work by firmly sequestering iron and preventing it from being used in cellular activities, resulting in a decrease in intracellular iron levels and a slowing of cellular metabolism. Iron chelators have been shown to reduce chemoresistance and the possibility of tumor cell progression by inhibiting the expression of stemness-related markers such as OCT4, SOX2, KLF4, NANOG, and SALL4 [22]. Iron chelators have also been shown to block DNA and histone demethylation enzymes including TET enzymes and JmjC domain-containing proteins, which are iron-dependent oxygenases involved in transcriptional control and DNA repair [23, 24]. Numerous Food and Drug Administration (FDA)-approved iron chelators, such as deferoxamine (DFO), deferiprione (DFP), and deferasirox (DFX), in vitro have been promising in cancers, including leukemia, hepatoma, neuroblastoma, prostate, and breast cancer [25–30]. Some compounds with iron-chelating properties have been reported to be used for cancer therapy. Di-2-pyridylketone-4,4,-dimethyl-3-thiosemicrbazone (Dp44mT) exhibits anti-proliferative activity both in vitro and in vivo in models of pancreatic, neuroblastoma, and lung cancers [31–33]. Curcumin, derived from the plant Curcuma longa, has also iron-chelating ability and shows efficacy against colon, duodenal, stomach, esophageal, and oral cancers [34]. However, iron chelation treatment has been not yet approved for clinical cancer treatment, due to dose-limiting toxicities, lack of tumor cell selectivity, and ineffective intratumoral concentrations [35–38].

Targeting iron metabolism-related proteins has been developed into a promising avenue of cancer therapy. Although several clinical trials have been conducted on antibodies against TFR1 and show anti-tumor efficacy, immunogenicity of therapeutic protein remains a major concern directly related to patient safety [39]. TFR1-targeted delivery systems could circumvent systemic toxicity, and preliminary results are promising. MBP-426, a Tf-conjugated oxaliplatin-liposome, treats gastric and esophageal adenocarcinomas in a phase II clinical trial (NCT00964080). SGT-53 and SGT-94, targeting to TFR on cancer cells via a TFR1-scFv to deliver the plasmid DNA into cells, are currently under clinical evaluation (NCT02340117, NCT01517464). In addition to TFR1, targeting downregulation of ferritin light chain (FTL) protein by miR-133a increased sensitivity of breast cancer cells to chemotherapy drugs [40]. Although cancer therapies targeted at iron metabolism have manifested a certain level of success in some malignancies, the overall effectiveness is far from satisfactory partly due to adaptive resistance of CSCs.

### Iron-buffering abilities of CSCs in therapy resistance

Abnormal metabolism of iron in CSCs reveals the direct driving force of CSC tumorigenicity and therapeutic resistance. Due to adaptive resistance of CSCs, recent studies suggest CSCs possess iron-buffering capacity to maintain iron homeostasis under iron deficiency. It is reported that glioblastoma and breast CSCs express higher levels of TFR1 than non-CSCs [3, 4]. During incubation with iron chelator, glioblastoma CSCs could increase iron uptake by upregulating TFR1, thus responding to iron deficiency [3]. Breast CSCs trigger the degradation of ferritin into lysosomes replying to iron depletion [4]. In addition, stem cell marker has been demonstrated as an alternative pathway in iron acquisition of CSCs by mediating iron endocytosis. CD133 is regulated by iron and its expression has been linked to endocytosis of Tf/TFR1 or iron uptake suggesting the existence of CD133-Tf-iron network [15]. CD44 itself is transcriptionally regulated by mediating iron endocytosis, suggesting this pathway represents a powerful alternative to maintain iron homeostasis of CSCs [41]. These results demonstrate that the higher needs of iron in CSCs might trigger iron endocytosis pathway to maintain basal level of cellular iron (Fig. 1). Moreover, treatment of breast cancer cells with the iron chelator DFO might cause epigenetic reprogramming and decrease the expression of histone demethylases [42]. CpG island methylation is exhibited in the FPN1 gene promoter; hypermethylation of FPN1 promoter results in decreased FPN1 expression in basal breast cancer cells [43]. However, epigenetic reprogramming of CSCs in maintaining iron homeostasis remains to be clearly defined.

In the recent study, a small-molecule chelator of iron, fluorescent labeling of a clickable surrogate of deferoxamine (cDFO), provides a means to target the nucleus iron of breast CSCs, suggesting iron homeostasis of CSCs could be targeted at the nucleus using small-molecule inhibitors [41]. Therefore, novel small molecule iron-scavenging agents are urgently required to be discovered and therapy resistance of CSC-driven tumors needs to be resolved. Treatment of cancer cells with iron chelator induces increased accumulation of HIF-1 replying to iron deficiency [44, 45]. Lang et al. found that Tfr1-targeting liposomes could be employed to deliver DFO and the HIF1a inhibitor YC1 to target pancreatic cancer cells [46]. HIF-1, as a vital molecule for maintenance of CSCs, participates in cancer recurrence, metastasis, and therapy resistance [47]. In addition, Imran ul-haq et al. found that hyperbranched polyglycerol (HPG)-conjugated DFO, which is nanopolymer-based chelating system, exhibited nontoxicity and longer half-life, but was not tested in tumors [48]. This co-delivery nanoparticle system and combination therapy lay the foundation for iron chelation of CSCs in future. Given that abnormal metabolism of iron in CSCs provides the buffer capacity in resisting, it is unlikely that current iron chelation therapy will be effective in CSC-driven cancers. Further understanding of resistant mechanisms of CSCs to iron chelation leads to the development of promising treatments that target the ability of a cell to control iron metabolism. Potential treatment options against regulators of iron metabolism, including small-molecule inhibitors and nanotechnology platforms, to some extent, exert their antitumor effects in CSC-driven tumors.

### New therapeutic strategies to tackle iron-adaptive CSCs

High levels of free iron in cancer cells are associated with increased levels of the ROS production via Fenton reaction, which are more susceptible to lipid peroxidation-induced ferroptosis [49]. Ferroptosis is a specific type of cell death with distinct biochemical and genetic characteristics and differs from other forms of regulated cell death, including apoptosis, autophagy, and necrosis [50]. Since ferroptosis was discovered in 2012 as a novel iron-dependent type of regulated cell death triggered by ROS-induced lipid peroxidation, it is gaining a lot of attention as an important cell death pathway in various cancers [51]. However, CSCs possess nature resistance of rendering them sensitive to ferroptosis [52] (Fig. 1). Increased expression of ferritin in CSCs restrains ferroptosis by limiting the expansion of free iron pool, which might be protective mechanism against ferroptosis [53]. Mai et al. inhibits breast CSCs by salinomycin, which could induce the lysosomal degradation of ferritin and activate ferroptosis [4]. Recent studies have found there is the correlation between NRF2 expression and cell protection against ROS-induced ferroptosis [54]. CSC markers, including CD44 and ALDH, are found to be associated with elevated NRF2 expression, which suggests that NRF2 is a sensible target for eliminating CSCs via ferroptosis [55, 56]. In the lipid peroxidation process, lipid hydroperoxides are important intermediates. The glutathione peroxidase 4 (GPX4) transforms lipid hydroperoxides to lipid alcohols, which avoids the generation of iron-dependent lipid ROS. Dysfunctions in cysteine metabolism influence GPX4, and inhibiting GPX4 activity causes lipid peroxidation, which can lead to ferroptosis [57]. Cystine is uptaken by Xc^−^ system for the synthesis of glutathione, which further enhances the anti-ferroptosis activity of GPX4. The expression of CD44 variant isoform (CD44v) suppresses ferroptosis by interacting with Xc^−^ system [58]. The results from studies concluded that interference with GPX4 pathways results in ferroptotic death in CSCs, which presents as a novel strategy to counteract tumor relapse [59, 60]. CD44v-targeting drugs like salazosulfapyridine, whose effect is considered to be ROS production, are still in phase I trials for gastric cancer and non-small-cell lung cancer, suggesting that CSCs have dual capabilities, not only to scavenge ROS but also even in the presence of ROS, to exploit it to enhance CSC activity [61, 62]. As a result, the therapy of ROS-inducible CSCs by blocking anti-oxidant molecules like Cys and Xc-system should be more deeply considered. Understanding the mechanistic regulation of ferroptosis could open an additional opportunity to target CSCs.

**Fig. 2:**
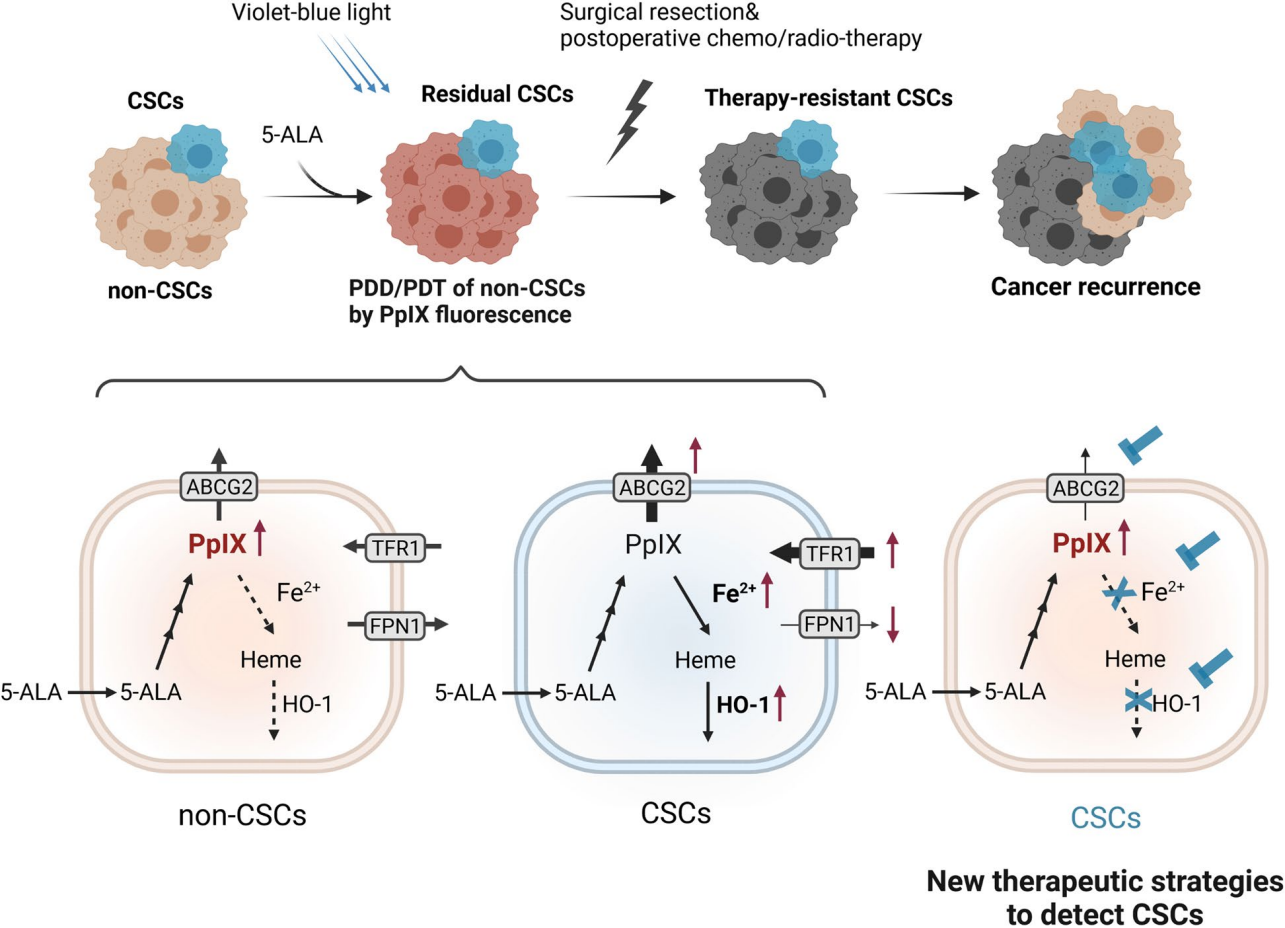
The current limitations and new therapeutic strategies of 5-ALA-based PDD/PDT to detect CSCs for cancer eradication. CSCs exhibit lower PpIX accumulation in 5-ALA-based PDD/PDT, potentially due to PpIX efflux by ABCG2 transporter, accelerated iron usage to convert PpIX to heme and the increased expression of HO-1 to accelerate PpIX-heme metabolism. The combination of 5-ALA-PDD/PDT with targeted proteins in heme/iron metabolism might establish the novel therapeutic strategies to eradicate CSCs. ABCG2, ATP-binding cassette sub-family G member 2; 5-ALA, 5-aminolevulinic acid; FPN1, ferroportin1; PDD, photodynamic diagnosis; PDT, photodynamic therapy; PpIX, protoporphyrin IX; TFR1, transferrin receptor 1; HO-1, heme oxygenase-1

Photodynamic diagnosis (PDD) and therapy (PDT), which utilize photosensitizers for fluorescence detection or photochemical therapeutic strategy, are promising non/minimally invasive approaches for cancer diagnosis and treatment [63]. 5-ALA is a crucial precursor in the heme biosynthesis pathway, where it is converted into the photosensitizing intermediate protoporphyrin IX (PpIX). PpIX is predominantly accumulated in cancer cells following 5-ALA treatment compared to normal cells, providing the foundation for the use of 5-ALA-based PDD and PDT in cancers [64, 65]. 5-ALA-based PDD and PDT are commonly used in the treatment of skin tumors and also have been employed for treatment of various tumors, such as bladder cancer, gastric cancer, head and neck cancer, T cell leukemia, and malignant glioma [66]. However, due to insufficient and heterogeneous PpIX accumulation, non-dermatologic applications of 5-ALA-based PDD and PDT have not moved beyond intial clinical trials. Recent studies demonstrate CSCs accumulate less PpIX, and thus, they might be resistant to 5-ALA-PDT [5]. The crucial parameters associated with CSC resistance to 5-ALA-PDT are abnormal expression of major proteins in iron metabolism of CSCs (Fig. 2). High ATP-binding cassette sub-family G member 2 (ABCG2) expression plays a pivotal role in regulating PpIX accumulation, and CSCs are enriched in the ABCG2-high ALA-expelling population [67, 68]. Kawai et al. demonstrate that CSCs could be targeted by the combination of 5-ALA-PDD/PDT with an ABCG2 inhibitor Ko143 in the gastrointestinal cancer cell lines [69]. CSCs have high intracellular iron due to upregulation of TFR1 and downregulation of FPN1 [3, 4, 8–10], and iron is the substrate for conversion of PpIX to heme by ferrochelatase (FECH). Previously, we found that reduced amounts of iron by DFO lead to enhanced PpIX accumulation in glioma CSCs. Meanwhile, we also observed that HO-1 is upregulated in them, which might accelerate the PpIX/heme metabolic pathway, leading to the poor 5-ALA-mediated accumulation of PpIX. Moreover, high expression of HO-1 predicts human glioma malignancies [5]. Therefore, HO-1 could be a reasonable target to enhance 5-ALA-based PDD and PDT in glioblastoma CSCs. Moreover, we found there is no significant difference of FECH expression between GBM and normal tissues, and its expression is not correlated with the prognosis of GBM patients [5]. However, Fujishiro et al. found that there is a significant upregulation of FECH and ABCG2 in human GBM cell line A172-derived CSCs, suggesting that glioma CSCs could be targeted by the combination of 5-ALA-PDD/PDT with FECH or ABCG2 inhibitors [70]. These results indicate that mechanisms of CSC resistance to 5-ALA-PDT might be diverse depending on the types of tumor cells, and the combination of 5-ALA-PDD/PDT with targeted proteins in heme/iron metabolism might establish the novel therapeutic strategies to eradicate CSCs.

## Conclusion

In this review, we briefly summarize current knowledge on iron metabolism in CSCs and their resistance to conventional cancer therapies, as well as the potential treatment options targeting iron/heme metabolism, especially focusing on ferroptosis and 5-ALA-PDT, which provide important insights into therapeutic strategies targeting CSCs. Although our knowledge about resistant mechanisms of CSCs in view of iron metabolism needs to be improved, potential treatment options targeting iron homeostasis, including small-molecule inhibitors, nanotechnology platforms, ferroptosis, and 5-ALA-PDT, might be a focus of future research for development of innovative cancer therapies against CSCs.

## Data Availability

Further information and requests for resources and reagents should be directed to the authors: Tetsuya Taga (taga.scr@mri.tmd.ac.jp) and Kouichi Tabu (k-tabu.scr@mri.tmd.ac.jp).

## Abbreviations

ABCG2: ATP-binding cassette sub-family G member 2
5-ALA: 5-Aminolevulinic acid
ALDH: Aldehyde dehydrogenase
CD44v: CD44 variant isoform
cDFO: Clickable surrogate of deferoxamine
CSCs: Cancer stem cells
Cys: Cystine
DFO: Deferoxamine
DFP: Deferiprione
DFX: Deferasirox
Dp44mT: Di-2-pyridylketone-4,4,-dimethyl-3-thiosemicrbazone
DMT1: Divalent metal transporter 1
FDA: Food and Drug Administration
FECH: Ferrochelatase
FPN1: Ferroportin 1
FTL: Ferritin light chain
GPX4: Glutathione peroxidase 4
HO-1: Heme oxygenase-1
IREs: Iron-responsive elements
IRP2: Iron regulatory protein 2
LIPS: Labile iron pool
NRF2: Nuclear factor erythroid 2-related factor 2
PDD: Photodynamic diagnosis
PDT: Photodynamic therapy
PpIX: Protoporphyrin IX
ROS: Reactive oxygen species
STEAP3: Six-transmembrane epithelial antigen of the prostate3
System Xc-: Cystine/glutamic acid transporter
TF: Transferrin
TFR1: Transferrin receptor 1

## References

[1] Clarke MF, Dick JE, Dirks PB, Eaves CJ, Jamieson CH, Jones DL (2006). Cancer stem cells--perspectives on current status and future directions: AACR Workshop on cancer stem cells. Cancer Res.

[2] El Hout M, Dos Santos L, Hamaï A, Mehrpour M (2018). A promising new approach to cancer therapy: targeting iron metabolism in cancer stem cells. Semin Cancer Biol.

[3] Schonberg DL, Miller TE, Wu Q, Flavahan WA, Das NK, Hale JS (2015). Preferential iron trafficking characterizes glioblastoma stem-like cells. Cancer Cell.

[4] Mai TT, Hamai A, Hienzsch A, Caneque T, Muller S, Wicinski J (2017). Salinomycin kills cancer stem cells by sequestering iron in lysosomes. Nat Chem.

[5] Wang W, Tabu K, Hagiya Y, Sugiyama Y, Kokubu Y, Murota Y (2017). Enhancement of 5-aminolevulinic acid-based fluorescence detection of side population-defined glioma stem cells by iron chelation. Sci Rep.

[6] Recalcati S, Gammella E, Cairo G (2019). Dysregulation of iron metabolism in cancer stem cells. Free Radic Biol Med.

[7] Torti SV, Torti FM (2020). Iron and cancer: 2020 vision. Cancer Res.

[8] Rychtarcikova Z, Lettlova S, Tomkova V, Korenkova V, Langerova L, Simonova E (2017). Tumor-initiating cells of breast and prostate origin show alterations in the expression of genes related to iron metabolism. Oncotarget..

[9] Raggi C, Gammella E, Correnti M, Buratti P, Forti E, Andersen JB (2017). Dysregulation of iron metabolism in cholangiocarcinoma stem-like cells. Sci Rep.

[10] Lobello N, Biamonte F, Pisanu ME, Faniello MC, Jakopin Ž, Chiarella E (2016). Ferritin heavy chain is a negative regulator of ovarian cancer stem cell expansion and epithelial to mesenchymal transition. Oncotarget..

[11] Pan X, Lu Y, Cheng X, Wang J (2016). Hepcidin and ferroportin expression in breast cancer tissue and serum and their relationship with anemia. Curr Oncol.

[12] Chen Q, Wang L, Ma YC, Wu XN, Jin LY, Yu FL (2014). Increased hepcidin expression in non-small cell lung cancer tissue and serum is associated with clinical stage. Thorac Cancer.

[13] Kuhn LC (2015). Iron regulatory proteins and their role in controlling iron metabolism. Metallomics..

[14] O'Donnell KA, Yu D, Zeller KI, Kim JW, Racke F, Thomas-Tikhonenko A (2006). Activation of transferrin receptor 1 by c-Myc enhances cellular proliferation and tumorigenesis. Mol Cell Biol.

[15] Bourseau-Guilmain E, Griveau A, Benoit JP, Garcion E (2011). The importance of the stem cell marker prominin-1/CD133 in the uptake of transferrin and in iron metabolism in human colon cancer Caco-2 cells. PLoS One.

[16] Basuli D, Tesfay L, Deng Z, Paul B, Yamamoto Y, Ning G (2017). Iron addiction: a novel therapeutic target in ovarian cancer. Oncogene..

[17] Tam WL, Weinberg RA (2013). The epigenetics of epithelial-mesenchymal plasticity in cancer. Nat Med.

[18] Wainwright EN, Scaffidi P (2017). Epigenetics and cancer stem cells: unleashing, hijacking, and restricting cellular plasticity. Trends Cancer.

[19] Toh TB, Lim JJ, Chow EK (2017). Epigenetics in cancer stem cells. Mol Cancer.

[20] Torti SV, Torti FM (2013). Iron and cancer: more ore to be mined. Nat Rev Cancer.

[21] Torti SV, Manz DH, Paul BT, Blanchette-Farra N, Torti FM (2018). Iron and cancer. Annu Rev Nutr.

[22] Szymonik J, Wala K, Górnicki T, Saczko J, Pencakowski B, Kulbacka J (2021). The impact of iron chelators on the biology of cancer stem cells. Int J Mol Sci.

[23] Roatsch M, Hoffmann I, Abboud MI, Hancock RL, Tarhonskaya H, Hsu KF (2019). The clinically used iron chelator deferasirox is an inhibitor of epigenetic jumonjiC domain-containing histone demethylases. ACS Chem Biol.

[24] Sarno F, Papulino C, Franci G, Andersen JH, Cautain B, Melardo C (2018). 3-Chloro-N’-(2-hydroxybenzylidene) benzohydrazide: an LSD1-selective inhibitor and iron-chelating agent for anticancer therapy. Front Pharmacol.

[25] Heath JL, Weiss JM, Lavau CP, Wechsler DS (2013). Iron deprivation in cancer--potential therapeutic implications. Nutrients.

[26] Breccia M, Alimena G (2013). Efficacy and safety of deferasirox in myelodysplastic syndromes. Ann Hematol.

[27] Simoes RV, Veeraperumal S, Serganova IS, Kruchevsky N, Varshavsky J, Blasberg RG (2017). Inhibition of prostate cancer proliferation by Deferipron. NMR Biomed.

[28] Knickle A, Fernando W, Greenshields AL, Rupasinghe HPV, Hoskin DW (2018). Myricetin-inducedapoptosis of triple-negative breast cancer cells is mediated by the iron-dependent generation of reactive oxygen species from hydrogen peroxide. Food Chem Toxicol.

[29] Bajbouj K, Shafarin J, Hamad M (2018). High-dose deferoxamine treatment disrupts intracellular iron homeostasis, reduces growth, and induces apoptosis in metastatic and nonmetastatic breast cancer cell lines. Technol Cancer Res Treat.

[30] Mertens C, Akam EA, Rehwald C, Brune B, Tomat E, Jung M (2016). Intracellular iron chelation modulates the macrophage iron phenotype with consequences on tumor progression. PLoS One.

[31] Lovejoy DB, Sharp DM, Seebacher N, Obeidy P, Prichard T, Stefani C (2012). Novel second-generation di-2-pyridylketone thiosemicarbazones show synergism with standard chemotherapeutics and demonstrate potent activity against lung cancer xenografts after oral and intravenous administration *in vivo*. J Med Chem.

[32] Kovacevic Z, Chikhani S, Lovejoy DB, Richardson DR (2011). Novel thiosemicarbazone iron chelators induce up-regulation and phosphorylation of the metastasis suppressor N-myc down-stream regulated gene 1: a new strategy for the treatment of pancreatic cancer. Mol Pharmacol.

[33] Guo ZL, Richardson DR, Kalinowski DS, Kovacevic Z, Tan-Un KC, Chan GC (2016). The novel thiosemicarbazone, di-2-pyridylketone 4-cyclohexyl-4-methyl-3-thiosemicarbazone (DpC), inhibits neuroblastoma growth *in vitro* and *in vivo* via multiple mechanisms. J Hematol Oncol.

[34] Jiao Y, Wilkinson J, Di X, Wang W, Hatcher H, Kock ND (2009). Curcumin, a cancer chemopreventive and chemotherapeutic agent, is a biologically active iron chelator. Blood..

[35] Mobarra N, Shanaki M, Ehteram H, Nasiri H, Sahmani M, Saeidi M (2016). A review on iron chelators in treatment of iron overload syndromes. Int J Hematol Oncol Stem Cell Res.

[36] Ceci A, Baiardi P, Felisi M, Cappellini MD, Carnelli V, De Sanctis V (2002). The safety and effectiveness of deferiprone in a large-scale, 3-year study in Italian patients. Br J Haematol.

[37] Saeki I, Yamamoto N, Yamasaki T, Takami T, Maeda M, Fujisawa K (2016). Effects of an oral iron chelator, deferasirox, on advanced hepatocellular carcinoma. World J Gastroenterol.

[38] Donfrancesco A, Deb G, Dominici C, Pileggi D, Castello MA, Helson L (1990). Effects of a single course of deferoxamine in neuroblastoma patients. Cancer Res.

[39] Daniels-Wells TR, Penichet ML (2016). Transferrin receptor 1: a target for antibody-mediated cancer therapy. Immunotherapy..

[40] Chekhun VF, Lukyanova NY, Burlaka CA, Bezdenezhnykh NA, Shpyleva SI, Tryndyak VP (2013). Iron metabolism disturbances in the MCF-7 human breast cancer cells with acquired resistance to doxorubicin and cisplatin. Int J Oncol.

[41] Muller S, Sindikubwabo F, Caneque T, Lafon A, Versini A, Lombard B (2020). CD44 regulates epigenetic plasticity by mediating iron endocytosis. Nat Chem.

[42] Brown RAM, Richardson KL, Kabir TD, Trinder D, Ganss R, Leedman PJ (2020). Altered iron metabolism and impact in cancer biology, metastasis and immunology. Front Oncol.

[43] Chen Y, Zhang S, Wang X, Guo W, Wang L, Zhang D (2015). Disordered signaling governing ferroportin transcription favors breast cancer growth. Cell Signal.

[44] Woo KJ, Lee TJ, Park JW, Kwon TK (2006). Desferrioxamine, an iron chelator, enhances HIF-1α accumulation via cyclooxygenase-2 signaling pathway. Biochem Biophys Res Commun.

[45] Ohara T, Noma K, Urano S, Watanabe S, Nishitani S, Tomono Y (2013). A novel synergistic effect of iron depletion on antiangiogenic cancer therapy. Int J Cancer.

[46] Lang J, Zhao X, Wang X, Zhao Y, Li Y, Zhao R (2019). Targeted co-delivery of the iron chelator deferoxamine and a HIF1α inhibitor impairs pancreatic tumor growth. ACS Nano.

[47] Zhang Q, Han Z, Zhu Y, Chen J, Li W (2021). Role of hypoxia inducible factor-1 in cancer stem cells. Mol Med Rep.

[48] Imran ul-haq M, Hamilton JL, Lai BF, Shenoi RA, Horte S, Constantinescu I (2013). Design of long circulating nontoxic dendritic polymers for the removal of iron *in vivo*. ACS Nano.

[49] Hassannia B, Vandenabeele P, Vanden BT (2019). Targeting Ferroptosis to iron out cancer. Cancer Cell.

[50] Dixon SJ, Stockwell BR (2019). The hallmarks of ferroptosis. Ann Rev Cancer Biol.

[51] Yu H, Guo P, Xie X, Wang Y, Chen G (2017). Ferroptosis, a new form of cell death, and its relationships with tumourous diseases. J Cell Mol Med.

[52] Elgendy SM, Alyammahi SK, Alhamad DW, Abdin SM, Omar HA (2020). Ferroptosis: an emerging approach for targeting cancer stem cells and drug resistance. Crit Rev Oncol Hematol.

[53] Battaglia AM, Chirillo R, Aversa I, Sacco A, Costanzo F, Biamonte F (2020). Ferroptosis and cancer: mitochondria meet the “iron maiden” cell death. Cells..

[54] Kahroba H, Shirmohamadi M, Hejazi MS, Samadi N (2019). The Role of Nrf2 signaling in cancer stem cells: from stemness and self-renewal to tumorigenesis and chemoresistance. Life Sci.

[55] Kim D, Choi BH, Ryoo IG, Kwak MK (2018). High NRF2 level mediates cancer stem cell-like properties of aldehyde dehydrogenase (ALDH)-high ovarian cancer cells: inhibitory role of all-trans retinoic acid in ALDH/NRF2 signaling. Cell Death Dis.

[56] Ryoo IG, Choi BH, Ku SK, Kwak MK (2018). High CD44 expression mediates p62-associated NFE2L2/NRF2 activation in breast cancer stem cell-like cells: implications for cancer stem cell resistance. Redox Biol.

[57] Lewerenz J, Hewett SJ, Huang Y, Lambros M, Gout PW, Kalivas PW (2013). The cystine/glutamate antiporter system x(c)(-) in health and disease: from molecular mechanisms to novel therapeutic opportunities. Antioxid Redox Signal.

[58] Ishimoto T, Nagano O, Yae T, Tamada M, Motohara T, Oshima H (2011). CD44 variant regulates redox status in cancer cells by stabilizing the xCT subunit of system xc(-) and thereby promotes tumor growth. Cancer Cell.

[59] Hangauer MJ, Viswanathan VS, Ryan MJ, Bole D, Eaton JK, Matov A (2017). Drug-tolerant persister cancer cells are vulnerable to GPX4 inhibition. Nature..

[60] Seiler A, Schneider M, Förster H, Roth S, Wirth EK, Culmsee C (2008). Glutathione peroxidase 4 senses and translates oxidative stress into 12/15-lipoxygenase dependent- and AIF-mediated cell death. Cell Metab.

[61] Kohei S, Toshihiko D, Osamu N, Miki F, Hiromi H, Shogo N (2017). Phase I study of sulfasalazine and cisplatin for patients with CD44v-positive gastric cancer refractory to cisplatin (EPOC1407). Gastric Cancer.

[62] Kohei O, Kaname N, Chiyo KI, Hiroaki O, Akitaka F, Shinya S (2017). Phase I study of salazosulfapyridine in combination with cisplatin and pemetrexed for advanced non-small-cell lung cancer. Cancer Sci.

[63] Stummer W, Pichlmeier U, Meinel T, Wiestler OD, Zanella F, Reulen HJ (2006). Fluorescence-guided surgery with 5-aminolevulinic acid for resection of malignant glioma: a randomised controlled multicentre phase III trial. Lancet Oncol.

[64] Agostinis P, Berg K, Cengel KA, Foster TH, Girotti AW, Gollnick SO (2011). Photodynamic therapy of cancer: an update. CA Cancer J Clin.

[65] Nokes B, Apel M, Jones C, Brown G, Lang JE (2013). Aminolevulinic acid (ALA): photodynamic detection and potential therapeutic applications. J Surg Res.

[66] Casas A (2020). Clinical uses of 5-aminolaevulinic acid in photodynamic treatment and photodetection of cancer: a review. Cancer Lett.

[67] Koizumi N, Harada Y, Minamikawa T, Tanaka H, Otsuji E, Takamatsu T (2016). Recent advances in photodynamic diagnosis of gastric cancer using 5-aminolevulinic acid. World J Gastroenterol.

[68] Park CY, Tseng D, Weissman IL (2009). Cancer stem cell-directed therapies: recent data from the laboratory and clinic. Mol Ther.

[69] Kawai N, Hirohashi Y, Ebihara Y, Saito T, Murai A, Saito T (2019). ABCG2 expression is related to low 5-ALA photodynamic diagnosis (PDD) efficacy and cancer stem cell phenotype, and suppression of ABCG2 improves the efficacy of PDD. PLoS One.

[70] Fujishiro T, Nonoguchi N, Pavliukov M, Ohmura N, Kawabata S, Park Y (2018). 5-Aminolevulinic acid-mediated photodynamic therapy can target human glioma stem-like cells refractory to antineoplastic agents. Photodiagn Photodyn Ther.

